# A review of anaesthesia for emergency laparotomy in paediatric intestinal obstruction in Enugu, Nigeria

**Published:** 2009-10-16

**Authors:** Ugochukwu Vincent Okafor, Jerome Azike

**Affiliations:** 1 Department of Anaesthesia, University of Nigeria Teaching Hospital (UNTH), Enugu, Nigeria,; 2 Department of Surgery, Imo state University Teaching Hospital, Orlu, Imo state, Nigeria.

**Keywords:** Anaesthesia, Laparotomy, Children, Nigeria

## Abstract

**Background:**

To review the anaesthetic management and outcome for emergency laparotomy for paediatric intestinal obstruction in the University of Nigeria Teaching Hospital, Enugu, Nigeria.

**Methods:**

The anaesthetic charts and folders of pediatric patients that had emergency laparotomy for intestinal obstruction in the general operating theatre of the University of Nigeria Teaching Hospital (UNTH), Enugu, Nigeria, from October 2007 – September 2008 were reviewed. The records were examined for anaesthetic technique, patient primary diagnosis, intra-operative events, blood and fluid therapy and patient outcome. Patients above thirteen years were excluded.

**Results:**

Forty-four out of 285 (15.7%) paediatric patients underwent emergency laparotomy for intestinal obstruction in the general operating theatre. There were 29 males and 15 females. The average age of the patients was 3.75 years. There were a total of 1674 anesthetics in the general operating theatre during the study. The leading causes of intestinal obstruction in this study were typhoid peritonitis (14 or 31.8%), intussusceptions (14 or 31.8%) and congenital anomalies (11 or 25%). Six patients (13%) had a preoperative packed cell volume of less than 30%, while ten patients received intra-operative blood transfusion (21.7%). There was one anesthetic death to give a case mortality rate of 2.2%.

**Conclusion:**

The mortality rate in this study shows the importance and relevance of trained providers of anaesthesia managing paediatric patients in the developing world. Early presentation of patients allowed time for resuscitation and fewer complications before surgery.

## Background

The issue of provision of pediatric anaesthesia in the developing world has been a source of concern to the World Federation of Anesthetists (WFSA) [1]. This is because of the special skill needed in managing these patients as children are considered a high risk surgical population [2, 3]. While results for trained pediatric anesthetists are reportedly excellent [4], complications are more likely to occur with poorly trained providers of anesthesia [5]. In the developing world, lack of materials and skilled manpower has resulted in poorly trained providers of anaesthesia administering pediatric anesthesia with inadequate machinery [6, 7].

These challenges have been reduced in parts of West Africa like Nigeria by the training of specialist anesthetists by the National Postgraduate Medical College of Nigeria and the regional West African Postgraduate Medical College [7]. These specialists work in most of the tertiary care hospitals in the country. There is a paucity of literature on the role of the anaesthetist in the management of emergency paediatric intestinal obstruction. This is important because the outcome depends in part on the quality of anaesthesia. Thus, this study reviews the anesthetic management and outcome of pediatric patients presenting for emergency laparotomy for intestinal obstruction in our centre over a twelve month period.

## Method

### Study area

The anesthetic charts and folders of pediatric patients that had emergency exploratory laparotomy for intestinal obstruction at the general operating theatre of the University of Nigeria Teaching Hospital (UNTH), Enugu, Nigeria, from October 2007 – September 2008 were reviewed. The records were examined for anesthetic technique, patient primary diagnosis, blood and fluid therapy and patient outcome. Patients above thirteen years were excluded. Pediatric anesthesia in the hospital is predominantly provided by senior trainees known as Senior Registrars (trainees who have completed more 24 months posting including a pass in the part one fellowship examination of either the National Postgraduate Medical College of Nigeria or the West African Postgraduate Medical College). Neonatal anesthesia is usually provided by consultants or the most senior trainee specialists who have spent more than four years in training. The anesthesia department has five consultants.

The pediatric surgery department has three pediatric surgeons. In early 2007, the hospital moved to its permanent site where the theatre was well equipped by VAMED health care services, an Austrian health care service provider. They installed multi-channel monitors (pulse oximetry, non-invasive blood pressure monitor, temperature, electrocardiography and capnography), and modern anaesthetic machines with low flow systems. In addition to these, the precordial stethoscope is another important monitor. Drugs avaliable for use in pediatric anesthesia include ketamine, sodium thiopentone, propofol (induction agents), suxamethonium, pancuronium and atracurium (neuromuscular blockers). Opiates include fentanyl, morphine, pethidine, tramadol and pentazocine. Glycopyrollate and atropine are the available anticholinergics and diazepam is the commonly used oral/parenteral anxiolytic. Available volatile agents are halothane and isoflurane.

Oxygen and medical air provide the fresh gas flow. The formula for fluid therapy is shown in table two. For third space losses; an extra 3–5mls/kg body weight per hour is added. Hypotonic and isotonic solutions used are 4.3%/0.18% saline, half strength Darrows’ solution, normal saline and Hartmann’s solution. The American Society of Anaesthetists’ (ASA) classification was used to grade the physical status of the patients.

## Results

There were 44 pediatric anaesthetics for emergency exploratory laparotomy out of a total of 1674 anesthetics in the general operating theatre. Two hundred and eighty-five were pediatric patients (17% of all operations).There were 29 males and 15 females (approximate ratio 2:1). The average age of the patients was 3.75 years (range from 2 days to 13 years). There were 4 neonates (< 28 days) at 8.7%. Table 1 shows the causes of intestinal obstruction. The leading causes of intestinal obstruction in this study were typhoid peritonitis (14 or 31.8%), intussusception (14 or 31.8%) and congenital anomalies (11 or 25%). Fifteen children were considered to be ASA two patients, 23 patients; ASA 3 and 6 patients; ASA 4. Six patients (13%) had a preoperative packed cell volume of less than 30%, while ten patients received intra-operative blood transfusion (21.7%). All the patients received controlled ventilation except one case where both controlled and spontaneous respiration was used for maintenance. Jackson Ree’s modification of the Ayre’s T- piece was used in a majority of the patients and mechanical ventilation with close circuit, low flow system in a minority, especially the older children. There was one anesthetic death in a patient with typhoid perforation and septicemia to give a case mortality rate of 2.2%, while another patient that presented with bleeding typhoid perforation was managed postoperatively in the intensive care unit (ICU).

## Discussion

The 44 pediatric patients that presented for exploratory laparotomy in this study represented 15.7% of all pediatric surgeries during this study period and 2.6% of all anesthetics in the general operating theatre during the study period. Two hundred and eighty-five anaesthetics were administered to children under 13 years of age (17% of total anesthetics). This figure is higher than the figure of 12% from Togo, West Africa [8] and the 6% for under −10 _s_ in Chad in Central Africa [9]. The 24% abdominal surgery rate in this study is lower than the 37.5% in a study from neighboring Cameroon [10]. It has been widely reported that intestinal obstruction is a relatively common indication for laparotomy in children [11]. This calls for prudent preoperative fluid therapy because of the attendant dehydration and hypovolemia. The leading causes of intestinal obstruction in this study were typhoid peritonitis, intussusception and congenital anomalies. All are significant causes of paediatric intestinal obstruction [12, 13].

Because of prior resuscitation before surgery, most of our patients come to the theatre with an intravenous line in place. In cases without a line, a ketamine/diazepam cocktail is given to induce amnesia before intravenous access is achieved. This is due to the unavailability of the local anaesthetic creams, even though they are relatively inexpensive. Though, there are CVP (central venous pressure) monitors, their use is mainly restricted to cardiac surgery. Non-invasive blood pressure monitors, oximeter pulse waveform and capillary refill are used in assessing haemodynamic status, together with the pulse volume, especially in older children. The anaesthetic technique depends on the skill and choice of the anaesthetist. As in all cases of full stomach, rapid sequence intubation is used. Ketamine is a preferred analgesic because of its potency and the fact that it does not depress respiration. Fentanyl is popular with older children and naloxone has been recently procured. Atracurium, pancuronium, halothane/isoflurane, opiates, ketamine and oxygen with medical air were used for maintenance. We prefer extubating our patients awake. Postoperatively, we give supplementary oxygen to patients with abdominal distension because of the diaphragmatic splinting caused by the distended abdomen. Tramadol may be used in the immediate postoperative period as it is less likely to depress respiration while providing analgesia which aids expectoration.

The patients received half strength Darrow’s solution and/or infusions of 4.3% dextrose/0.18% saline intraoperatively. There is still debate in the literature whether the use of these fluids is appropriate in children as they reportedly cause hyponatremia [14–16]. Inspite of this controversy, it was reported from a survey that 20–25% of anaesthetists and 36–39% of paediatric surgeons in Britain use dextrose 4.3%/saline 0.18% for surgical patients [17]. Significantly, a British report by the Medical Control Agency/Committee on safety of Medicines and by the Joint Royal Colleges of Paediatrics and Child Health (RCPCH)/Neonatal and Paediatric Pharmacists’ Standing Committee on Medicines in 2002 concluded that the problem was an issue of clinical practice rather than product regulation and that the use of Dextrose 4.3%/saline 0.18% could be continued albeit carefully in the postoperative period [18]. In our centre, fluids used in the wards are prescribed by the surgeons, while the anaesthetists prescribe the fluids used intraoperatively.

The pediatric surgeons get the patients prepared for surgery by ordering the necessary investigations including the preoperative resuscitation of the patients. This includes the use of normal saline, half strength Darrow’s solution and/or infusions of 4.3% dextrose/0.18% saline depending on the clinical scenario. Because these cases rarely involved bleeding, there were some hours for the patients to be resuscitated. In patients that presented after office hours, usually referred from peripherial hospitals, it was more difficult getting the electrolyte profile. Six patients presented with electrolyte imbalance, mainly increased urea, creatinine, potassium and low bicarbonate levels which were corrected perioperatively.

Ten patients received blood transfusion intra-operatively when the estimated blood loss was 10% or more of the body weight. There was one death in this study giving a case mortality rate of 2.2%. This mortality rate is higher than that reported from the United Kingdom, which is less than 1% [19]. It is however lower than the reported 5–11% mortality rates from India [20] and another Nigerian study at 11% [21]. An earlier study on typhoid ileal perforation in our centre (1995–2004) reported a 19% mortality rate [22]. The relatively low mortality rate in this study may be the result of patients presenting earlier than before, adequate monitoring and good pre-operative preparations by the surgeons. The improved economic climate due to increased price of crude oil (Nigeria is the world’s sixth largest producer of crude oil) meant that more parents could afford the cost of medical care and transportation services. The improved quality of anaesthesia played a pivotal role as the outcome of surgery is often tied to the quality of anaesthesia. The fact that the patients were managed by senior doctors could be another contributory factor. The availability of generic brands of potent parenteral antibiotics may have helped in reducing the mortality rate [23]. The lone death occurred in a 6 year old who presented in extremis with typhoid peritonitis and septicemia (presented > one week after the onset of symptoms). The time of presentation is an accepted prognostic factor in intestinal obstruction [20, 24]. The deceased patient was managed by senior trainee specialists.

The importance of this study for Sub-saharan Africa is that governments can train a few specialists in Africa or overseas who will come back and train others on the safe delivery of paediatric anaesthesia. Their services will be invaluable in rural hospitals. In more resource poor settings, ketamine-midazolam/diazepam-atracurium/pancuronium with controlled ventilation using oxygen and medical air can be used safely. Oxygen is fairly cheap in Nigeria [23]. Generic brands of glycopyrollate are available and can be used with ketamine (to dry secretions) without the great increase in the pulse rate associated with atropine use. Monitoring urine output will help in assessing global tissue perfusion. Monitors like the pulse oximeter would be a great asset. This is another area where the WFSA can assist by donating fairly used oximeters to developing nations. Governments in developing nations can help by investing more in their people through programmes like health insurance schemes, especially for children and mothers.

## Conclusion

The mortality rate in this study shows the importance and relevance of trained providers of anaesthesia managing paediatric patients in a developing country. Early presentation of patients allowed time for resuscitation and fewer complications before surgery.

## Figures and Tables

**Table 1:** Indications for surgery (n=44)

** Surgical diagnosis **	** Number (n=44) **
Intussusception	14 (31.8%)
Typhoid peritonitis	14 (31.8%)
Congenital anomalies	11(25%)
Postoperative intestinal obstruction	3 (6.8%)
Adhesive intestinal obstruction	1 (2.3%)
Peritonitis due to ruptured appendix	1 (2.3%)
